# Improving Curcumin Bioavailability: Current Strategies and Future Perspectives

**DOI:** 10.3390/pharmaceutics13101715

**Published:** 2021-10-17

**Authors:** Rita Tabanelli, Simone Brogi, Vincenzo Calderone

**Affiliations:** Department of Pharmacy, University of Pisa, Via Bonanno 6, I-56126 Pisa, Italy; ritatabanelli@gmail.com (R.T.); vincenzo.calderone@unipi.it (V.C.)

**Keywords:** curcumin, delivery methods, nanoparticles, delivery by design, bioavailability

## Abstract

Curcumin possesses a plethora of interesting pharmacological effects. Unfortunately, it is also characterized by problematic drug delivery and scarce bioavailability, representing the main problem related to the use of this compound. Poor absorption, fast metabolism, and rapid systemic clearance are the most important factors contributing to low curcumin levels in plasma and tissues. Accordingly, to overcome these issues, numerous strategies have been proposed and are investigated in this article. Due to advances in the drug delivery field, we describe here the most promising strategies for increasing curcumin bioavailability, including the use of adjuvant, complexed/encapsulated curcumin, specific curcumin formulations, and curcumin nanoparticles. We analyze current strategies, already available in the market, and the most advanced technologies that can offer a future perspective for effective curcumin formulations. We focus the attention on the effectiveness of curcumin-based formulations in clinical trials, providing a comprehensive summary. Clinical trial results, employing various delivery methods for curcumin, showed that improved bioavailability corresponds to increased therapeutic efficacy. Furthermore, advances in the field of nanoparticles hold great promise for developing curcumin-based complexes as effective therapeutic agents. Summarizing, suitable delivery methods for this polyphenol will ensure the possibility of using curcumin-derived formulations in clinical practice as preventive and disease-modifying therapeutics.

## 1. Introduction

Finding appropriate delivery systems is a challenging task to utilize natural products as effective treatments for a given disease. Considering that many natural products have interesting pharmacological effects and that they can represent a valuable source for developing innovative drugs [1,2], currently, natural compounds of interest are under investigation for improving their bioavailability by employing different technologies [3,4,5]. Natural products are not chemically stable and are susceptible to oxidative degradation, affecting the integrity of molecules and leading to the development of free radicals. Additionally, it is difficult to employ natural products in formulations due to unfavorable features, including low solubility, inadequate bioavailability, and weak stability in front of environmental stresses (e.g., exposure to oxygen and light, high temperature, and pH) [6]. Consequently, to maintain the peculiar profile of these compounds, specific methods of delivery offer the possibility to use natural compounds for treating human disorders. In this review article, we examine the most advanced delivery approaches for enhancing curcumin bioavailability, considering the technologies used and clinical trials that prove the effectiveness of the methods. Curcumin holds great promise in clinical practice since it behaves as a multifunctional ligand possessing a plethora of pharmacological effects, targeting several cellular pathways [7,8]. According to the multifactorial and heterogeneous nature of different disorders, including inflammatory, metabolic, neoplastic, neurodegenerative, and central nervous system (CNS)-related disorders, compounds with no side effects, that are non-toxic to patients, and have multiple properties, such as curcumin, are excellent candidates for treating these pathologies [9,10]. This strategy offers a better chance of effective prophylaxis or treatment and supports the ongoing research and development of curcumin as a preventive and disease-modifying agent [11]. These aspects have been reviewed considering clinical trials performed by enrolling healthy people to assess the enhancement of curcumin bioavailability by exploring different formulations. Furthermore, we discuss clinical outcomes using diverse curcumin formulations for treating several disorders (e.g., nonalcoholic fatty liver disease (NAFLD), knee osteoarthritis (OA), moderate hyperlipidemia metabolic syndrome risk, glioblastoma, obesity, impaired glucose tolerance or non-insulin-dependent diabetes mellitus, delayed onset muscle soreness (DOMS) and related muscle damage, osteo-muscular pain, chronic diabetic macular edema, advanced or metastatic pancreatic cancer).

## 2. From the Kitchen to the Bench

Curcumin (IUPAC: (1*E*,6*E*)-1,7-*bis*(4-hydroxy-3-methoxyphenyl)-1,6-heptadiene-3,5-dione), a polyphenol extracted from turmeric *Curcuma longa* L., was used from ancient times in traditional and ayurvedic medicine, especially in India and China [12]. Furthermore, for over 2000 years, the rhizome of turmeric has been employed in Asian cuisine, cosmetics, and fabric dyeing [13]. The main component of turmeric, curcumin, has been demonstrated to possess a plethora of interesting pharmacological effects, including anti-inflammatory, antioxidant, neuroprotective, chemopreventive, and chemotherapeutic activity [14,15]. Anti-inflammatory effects of curcumin were observed in the acute carrageenan-induced edema test in mice and rats after oral administration. The oral doses required to produce an anti-inflammatory effect, however, were much higher than the doses that were necessary for intraperitoneal (i.p.) administration, giving a similar effect. Thus, the oral ED_50_ was 100.2 mg/kg in mice and 48.0 mg/kg in rats [14]. Several studies have been conducted on anti-inflammatory effects of curcumin, concluding that in mice, this polyphenol inhibited edema at doses between 50 and 200 mg/kg. A 50% reduction in edema was achieved with a dose of 48 mg/kg body weight, with curcumin nearly as effective as cortisone and phenylbutazone at similar doses. In rats, a lower dose of 20–80 mg/kg decreased paw edema and inflammation. Curcumin also inhibited formaldehyde-induced arthritis in rats at a dose of 40 mg/kg [16,17]. Regarding its antioxidant profile, a recent meta-analysis highlighted that the effective dose of curcumin to obtain such an effect is 645 mg/die [18]. The anticancer activity of curcumin has been recently reviewed by Tomeh and colleagues, who reported a comprehensive overview of clinical applications of curcumin for treating different tumors (e.g., benign prostatic hypertrophy, 1 g/day for 24 weeks; breast cancer, 0.5–8 g/day for 7 days plus docetaxel in a phase I clinical trial; chronic myeloid leukemia, 5 g three times daily for 6 weeks plus imatinib (400 mg twice daily); pancreatic cancer, in a phase II clinical trial, 8 g/day for 8 weeks; prostate cancer, in a randomized controlled trial, 3 g/day for 3 months as a supplement to radiotherapy) [19]. Finally, curcumin showed neuroprotective effects, considering CNS-related disorders (e.g., depression) and ageing-related diseases (Alzheimer’s and Parkinson’s diseases), at doses ranging from 40 mg/kg i.p. for depression (rat models) to several grams (500 mg twice daily—4 g daily) per day in clinical trials for Alzheimer’s disease [20,21,22].

**Figure 1:**
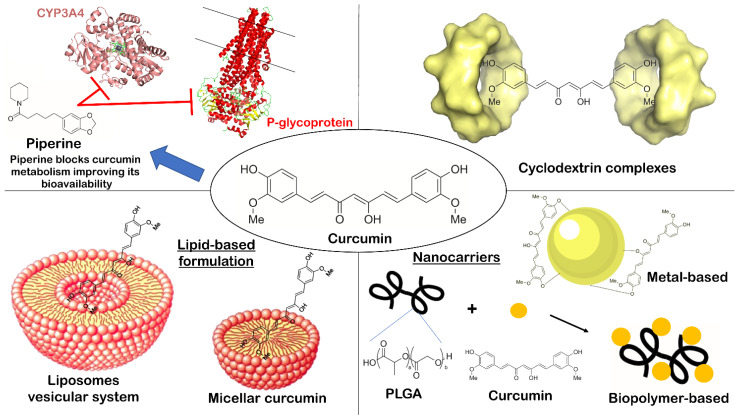
Schematic representation of the main strategies for improving the bioavailability of curcumin. CYP3A4 and P-glycoprotein were downloaded from protein data bank (PDB https://www.rcsb.org/; PDB IDs 7KVS [51] and 6C0V [52], respectively). Cyclodextrin structure was downloaded from The Cambridge Crystallographic Data Centre (CCDC https://www.ccdc.cam.ac.uk/; ID 762697; WEWTOJ) [53]. Structures were manipulated by PyMOL software (The PyMOL Molecular Graphics System, v1.8; Schrödinger, LLC, New York, 2015). Au–curcumin nanoparticle was designed according to Priyadarsini [46].

The beneficial properties of curcumin stem from the capacity to modulate many signaling pathways, such as survival pathways mediated by NF-kB, Akt, and growth factors, as well as cytoprotective pathways involving Nrf2, and metastatic and angiogenic pathways [23,24,25]. NF-kB modulates the expression of several chemokines and cytokines, including interleukins, interferons, lymphokines, and tumor necrosis factors (TNFs) [26]. Specifically, curcumin inhibits cytokine production by suppressing the NF-kB phosphorylation. By this specific targeting, curcumin regulates inflammatory processes and related diseases such as OA. It has been demonstrated that curcumin exerts protective effects on IL-1β-, TNFα-, or TNFβ-stimulated chondrocytes, indicating its chondroprotective properties for treating OA and related osteoarticular problems [27]. Accordingly, curcumin suppressed NF-kB pathway activation by repressing IkBα phosphorylation and degradation, as well as P65 nuclear translocation, attenuating inflammation [28,29,30]. Additionally, curcumin is a free radical scavenger with pro- and antioxidant activity [31]. Furthermore, curcumin binds metals, especially iron and copper, acting as an iron chelator [32]. Interestingly, in general, curcumin is well tolerated and safe, as several studies have confirmed low toxicity in humans (oral doses up to 12 g/die administered to adult subjects) [33,34,35,36]. On the other hand, other reports highlighted possible adverse effects, including DNA damage [37,38], time- and dose-dependent increase in reactive oxygen species (ROS) [39], and inhibition of p53 in colorectal cancer [40]. Moreover, also in clinical studies, some undesirable effects have been reported after employing higher dosage for 4 months (e.g., nausea, diarrhea, headache, rash, and yellow stool) [35,41]. Despite the encouraging pharmacological profile, curcumin suffers from several drawbacks, limiting its therapeutic potential. The problematic drug delivery and poor bioavailability are the main problems related to the use of this compound [42,43]. In fact, curcumin (logP 3.29 [44]) is almost insoluble in pure water (0.6 μg/mL), while an improvement of solubility was observed in organic solvents such as ethanol (1 mg/mL) [45,46]. Poor absorption, fast metabolism, and rapid systemic clearance are the most relevant factors that contribute to the low curcumin levels in plasma and tissues [43]. To overcome these issues, numerous strategies have been recently described, such as the use of adjuvants like piperine (interferes with curcumin metabolism), complexed/encapsulated curcumin, specific curcumin formulations, and curcumin nanoparticles (Figure 1) [47,48,49,50]. Accordingly, in the following sections, we report the main promising strategies for improving curcumin bioavailability that are supported by clinical trials, or in vivo studies, to provide an up-to-date overview of the technologies used and related evidence of success in human trials.

### 2.1. Ready-to-Go Strategies to Enhance Bioavailability

As a result of the broad spectrum of potential health benefits shown by curcumin, attempts to improve its bioavailability have been described over the years. Despite the high number of formulations tested, only some of them reached the market due to their unsuitability for commercial applications. In fact, many delivery formulations have been discontinued due to being inadequate for commercial purposes since they were too expensive, too difficult to produce, unstable under environmental conditions, or used ingredients not suitable for food applications [54,55]. This section examines commercial formulations, that overcame these concerns, for which human pharmacokinetic data are available. Formulations are presented according to the bioenhancement strategy, addressing the issues regarding the limited curcumin bioavailability. It is important to notice that the great variability within the clinical studies significantly affects the accurate comparison of the results [56]. Moreover, most of the available pharmacokinetic studies, analyzed the total curcumin content instead of free curcumin [57,58,59,60,61,62] since it was reported that blood samples were hydrolyzed with glucuronidase or sulfatase before the analysis. Due to the fact that curcumin undergoes extensive metabolism once orally administered, sulfate and glucuronide conjugates represent the predominant, although physiologically inactive, compounds. Indeed, many authors agreed that plasma-free curcumin represents the bioactive form of curcumin and is currently the best indicator of bioavailability and bioequivalence [63,64,65]. Therefore, enzymatic hydrolysis can lead to a misreading of the results with an over-estimation of the free, bioactive curcumin at least 10-fold higher compared to non-hydrolyzed plasma samples [66]. Thus, in this review, data resulting from sample hydrolysis will be defined as “total curcumin” compared to “free curcumin” from not-hydrolyzed samples.

#### 2.1.1. The Early Factors Impacting Metabolism

In Table 1, the pharmacokinetic profile of curcumin considering various curcumin-based preparations is reported. One of the first adopted strategies was to combine curcumin with the alkaloid piperine (inhibitor of UDP-glucuronosyltransferase and CYP3A4, and P-glycoprotein blocker). This approach showed a clear inhibition of curcumin metabolism [67,68]. The administration of 2 g of curcumin with 20 mg of piperine to ten healthy adult males, in a randomized crossover trial, resulted in a 20-fold increase in AUC compared with the administration of 2 g of pure curcumin alone [69]. Similarly, in a further work, the effect of co-administration of 2 g of curcumin and 5 mg of piperine (Bioperine^®^) [70] in six healthy subjects in a crossover-designed study was assessed. Three volunteers received only curcumin, while the remaining three received both the drug and the adjuvant. Results showed that the absorption of curcumin co-administered with piperine was approximately doubled [42]. However, more recently, the administration of 12 g of Curcumin C^3^ Complex^®^ [71] with 5 mg of Bioperine^®^ in an open-label, uncontrolled phase I pilot study, did not detect any trace of curcumin in blood samples of ten volunteers at any endpoint [72]. Recently, the efficacy of the formulation of curcumin + piperine has also been investigated in clinical practice. Panhai and colleagues evaluated the effect of co-administration of 500 mg of curcumin + 5 mg of piperine (Curcumin C^3^ Complex^®^ and Bioperine^®^, Sami Labs Ldt) in adult patients suffering from NAFLD. Seventy adults were arbitrarily assigned to the placebo or treatment group and consumed one capsule after meals for 12 weeks. Results showed that the administration of Curcumin C^3^ Complex^®^ and Bioperine^®^ improved liver and lipid profile without altering hematological parameters, thus reducing NAFLD severity compared to placebo [73]. The same research group also considered the effect of the formulation on serum inflammatory factors, namely, TNFα and IL-6, in a randomized double-blind placebo-controlled trial with 55 subjects with NAFLD. Dietary supplementation for 8 weeks resulted in a decrease in cytokine serum levels, suggesting their reduction can mediate the anti-steatotic effect of curcuminoids [74].

Another strategy that addresses the modulation of curcumin metabolism consists of the mixture of turmeric powder and turmeric essential oil. The latter, being particularly rich in turmerones, which act as p-glycoprotein inhibitors, increases intestinal permeability of curcumin [75]. BCM-95^®^CG is a patented [76], cost-effective formulation that relies on the synergism between sesquiterpenoids found in turmeric essential oil and curcuminoids. The mechanism hypothesized to underlie its efficacy considers a possible extra-intestinal absorption due to non-curcuminoid components of turmeric and a variation of the activity of phase II enzymes and P-glycoprotein due to its longer presence in blood [77,78]. Evaluation of BCM-95^®^CG (Biocurcumax™) oral bioavailability was obtained from a pilot crossover study conducted on 11 healthy human subjects. Volunteers, divided into three groups, were administered 2000 mg of BCM-95^®^CG and an equal dose of curcumin control or a formulation of curcumin–lecithin–piperine, respectively. Drugs were administered and then crossed over after a two-week washout period. Free curcumin AUC appeared to be 6.93 times higher in BCM-95^®^CG than that of curcumin control; furthermore, results suggested both early absorption and retained release of curcumin from BCM-95^®^CG compared to unformulated and curcumin-lecithin-piperine preparation [77]. Two clinical trials assessed the effectiveness of a curcumin—essential oil complex in the management of knee OA compared to common drugs, such as diclofenac or paracetamol. Together, these studies revealed that co-administration of 500 mg of BCM-95^®^CG plus 50 mg of diclofenac twice a day (group 1, 71 patients) compared to administration of 50 mg of diclofenac twice a day alone (group 2, 69 patients), as well as the administration of 500 mg of BCM-95^®^CG twice a day (73 patients) or 650 mg of paracetamol three times a day (71 patients), not only significantly improved knee OA symptoms, but reduced the gastrointestinal (GI) side effects related to NSAIDs treatment and the need of other analgesics, thus suggesting a good alternative treatment option [79,80].

#### 2.1.2. Solubility Enhancement as a Strategy to Improve Curcumin Bioavailability

Due to the fact that solubility enhancement is one of the major areas of interest in regards to curcumin dissolution, there are two main strategies to consider, the particle size reduction for increasing the surface area and the use of substances to improve curcumin dispersibility. Among them, surfactants, hydrophobic carriers, and the formation of inclusion complexes encompass the most used and successful approaches [56,86].

Novasol*^®^* (patented formulation by AQUANOVA AG) [87] is based on micellization: when Tween-80, a common surfactant, exceeds the crucial micellar concentration in aqueous solutions, micelles form and incorporate hydrophobic drugs, such as curcumin [56,86]. Micellar curcumin containing 7% curcumin powder and 93% Tween-80 has been tested on human healthy volunteers to assess its bioavailability by the same research group in at least three small clinical trials. In a single-blind crossover study, curcumin (410 mg) was administered to 23 healthy human volunteers as native powder or micellar curcumin after a 1-week washout period. Data based on total curcumin AUC showed a great increase in formulated curcumin bioavailability that appeared 185-fold higher compared to the unformulated powder [62]. Furthermore, they tested the efficacy of a lower dose of the aforesaid formulation: 80 mg of curcumin was given to 23 healthy subjects in a single-blind crossover trial. Administration of formulated and unformulated curcumin was separated by a 1-week washout. The total curcumin AUC was increased 88-fold compared to native curcumin, suggesting that curcumin bioavailability is affected by the administered dose [81]. Finally, Kocher and colleagues have deepened the efficacy of micellar curcumin in a randomized, double-blind crossover study involving much more moderately overweight and hyperlipidemic patients. Curcuminoids or placebo were administered with each principal meal for 6 weeks (curcumin dose = 241.2 mg/day) and then crossed over to the other regimen after 4 weeks of the washout phase. Although the total plasma curcumin concentration was higher compared to placebo and baseline samples after three and six weeks of treatment, the intervention did not show any alteration in blood lipid levels, inflammatory status, glucose, or iron homeostasis. The lack of effects could be ascribable to an insufficient tissue concentration for biological activity, longer intervention required, or more severely afflicted patients [50]. Novasol*^®^* efficacy was studied also on glioblastoma patients scheduled for surgery. Ten out of thirteen completed a study that consisted of drinking juice containing 1 g of micellar curcumin (corresponding to 57.4 mg curcumin) three times a day, for 4 days prior to surgery. Tissue analysis revealed an approximated intratumoral curcumin concentration of 0.15 μmol/L, being the highest ever found in a clinical study, although far from cytotoxic concentrations based on glioma cell culture models [88].

As mentioned, solubility issues might be overcome by curcumin inclusion in cyclodextrin complexes or by taking advantage of hydrophilic carriers such as polyvinyl pyrrolinone (PVP). Cyclodextrins consist of cyclic oligosaccharides formed by non-reducing chiral glucose-building blocks connected with a ring structure. Thanks to the hydrophilic portions of glucose units facing outwards, lipophilic cavity forms inside, allowing fat-soluble drugs to lodge. However, PVP consists of a water-soluble vector that increases curcumin water dispersibility [56,57,58]. Despite the abundance of in vitro and in vivo investigations, there have not been many clinical trials. One of these clinical studies was designed for testing the bioavailability of two innovative curcumin formulations, namely Cavacurmin*^®^* [89], a γ-cyclodextrin-based compound, and Curcuwin*^®^* [90], a combination of 63–75% PVP, 10–40% cellulosic derivatives, and 1–3% natural antioxidants. These formulations were both examined in two different randomized, double-blind trials and compared with unformulated standard curcumin and two other well-known curcumin associations. A single oral dose of formulated curcuminoids (376 mg) or standard curcumin (unformulated, 1800 mg) was administered to healthy human volunteers with 7 days of washout between doses. In both studies, twelve subjects out of fifteen completed the study. The total curcumin AUC resulted in 85-fold ad 136-fold greater in Cavacurmin*^®^* and Curcuwin*^®^*, respectively, compared to unformulated curcumin [57,58].

CurQfen*^®^* is a water-soluble curcumin formulation made of turmeric powder and fenugreek dietary fiber. The latter are rich in galactomannan units and form a non-digestible gel hydrocolloid that undergoes fermentation in the colon, thus protecting curcumin from degradation. Moreover, the association of curcumin and soluble fiber seems to prolong the drug release [56,66]. A randomized double-blind crossover study was completed on 50 subjects who were administered 1000 mg of CurQfen*^®^* (307 mg curcumin) or 411 mg of unformulated curcumin with similar content of curcuminoids. The two administrations were separated by a 10-day washout phase. The same group of subjects was tested also on a low-dose formulation—they were administered 250 mg of fenugreek formulation (76.7 mg of curcumin) or standard unformulated curcumin. Moreover, ten out of the fifty volunteers were selected to participate in an additional study. After the administration of a single oral dose of 1000 mg of CurQfen*^®^*, blood samples were withdrawn at specific time points and divided into two batches. Interestingly, for each sample, one half was treated with β-glucuronidase enzyme, while the other half was not. Beyond the dose dependency in the absorption of curcuminoids, CurQfen*^®^* significantly increased free curcumin oral bioavailability compared to the standard formulation. From the analysis of free and conjugated metabolites ratio emerged an AUC average enhancement of 17.21%, confirming the hydrolysis-related overestimation, and the ability of fenugreek formulation to mainly provide free unconjugated curcuminoids in plasma, probably due to curcumin protection against enzymatic activity [66]. CurQfen*^®^* effects were examined on 22 young obese men to test whether it could improve aortic stiffness and cardiovascular disease-associated blood biomarkers. Subjects were randomly administered 500 mg CurQfen*^®^* capsules (about 158 mg of curcumin) or placebo (fenugreek fibers) for 12 weeks. These two randomized double-blind studies confirmed a significant reduction in aortic stiffness related to inflammation markers decrease, while HDL and homocysteine improved significantly [91,92].

The particle size reduction increases the surface area, favoring rapid dissolution of curcumin [86]. Several patented curcumin formulations profit by this strategy to improve bioavailability. Among these, Biocurc*^®^* is a patented lipidic formulation [93] composed of curcumin (6.17% *w*/*w*), vitamin E (3.29% *w*/*w*), Labrasol (5.76% *w*/*w*), ethanol (8.23% *w*/*w*), Gelucire*^®^*44/14 (16.46% *w*/*w*), anhydrous citric acid (2.88% *w*/*w*), PEG 400 (55.55% *w*/*w*), and hydroxyl propyl cellulose (1.64% *w*/*w*) [82]. A preliminary open-label, randomized, parallel-design study evaluated the oral bioavailability of a single dose of formulated curcumin (750 mg) on 20 healthy male subjects. Despite the fact that plasma curcumin levels are relatively low, pharmacokinetic modeling revealed that curcumin from the formulation rapidly moves from the central circulation to peripheral tissues, providing an explanation of its therapeutic efficacy [82]. A randomized, double-blind, crossover study was executed considering 12 healthy adult subjects to compare the bioavailability of a single oral dose of Biocurc*^®^* (76 mg of total curcuminoids, of which 64.6 mg was curcumin) with unformulated curcumin (380 mg of total curcuminoids, of which 323 mg was curcumin, 95%). The samples were tested for curcumin glucuronide, curcumin sulfate, and free curcumin. While free curcumin from unformulated powder stayed near the baseline during the entire test period, free curcumin from Biocurc*^®^* showed at least two peaks, while total curcumin AUC appeared to be 94 times higher for the product than for the unformulated curcumin [63]. Particle size reduction is also a strategy that underlies Theracurmin*^®^* technology [94]. Theracurmin*^®^* consists of a nanoparticle colloidal dispersion, made of 10 *w*/*w* % curcumin, 2% other curcuminoids, 4% gum ghatti, 38% water, and 46% glycerin. Fourteen healthy volunteers were enrolled in a randomized clinical trial to test Theracurmin*^®^* oral bioavailability; 30 mg of the formulation or unformulated powder were given as a single oral dose to the subjects. Total curcumin AUC showed a 27-fold increase compared to unformulated curcumin [61]. The same researchers also evaluated the oral bioavailability of commercial curcumin beverages and compared it with a beverage containing Theracurmin*^®^*. A double-blind, single-dose, four-way crossover study involving 24 healthy volunteers was conducted. They were separated into four groups, each receiving four curcumin beverages, one every 7 days. Beverages were slightly different in composition, containing 30 or 40 mg of curcumin per 100 mL. For each beverage, pharmacokinetic results indicated that total curcumin was already detectable 30 min after ingestion, but peak plasma concentrations were undetectable for any preparation. This occurrence indicates a substantial involvement of other food ingredients in the beverage, and underling the role of food intake in curcumin oral bioavailability. Total curcumin AUC values from Theracurmin*^®^* preparation became about 1.5- to 4-fold higher than those of other formulations [59]. Theracurmin*^®^* efficacy was investigated in a double-blind placebo-controlled parallel-group randomized trial in patients with impaired glucose tolerance or non-insulin-dependent diabetes mellitus. Thirty-three patients were separated into two groups, one (*n* = 18) receiving placebo, the other (*n* = 15) receiving Theracurmin*^®^* (180 mg/day per for 6 months). After six months of screening, subjects in the placebo group displayed a considerable increase in oxidized LDL level, while both triglycerides (TG) and γ-GTP diminished in the Theracurmin*^®^* group [95]. In a 6-month open-label perspective study, 45 patients affected by knee OA were administered Theracurmin*^®^* (180 mg/day for 6 months) to test its clinical efficacy and safety. Thirteen out of 45 patients were treated with only Theracurmin*^®^*, while other patients were allowed other combined therapies, such as NSAIDs, pain relief patches, and hyaluronic acid knee injection treatment. Thirty-four patients were effective cases (75.6%), in which the treatment was effective, and the scores improved in at least one assessment within the 6 months. In contrast, 11 patients were not effective. The Theracurmin*^®^*-only group, included 10 effective (76.9%) and 3 not-effective cases [96].

#### 2.1.3. How to Improve Absorption Affecting Intestinal Uptake

Cureit^®^ is a new formulation from Aurea Biolabs [97], established on the recreation of the natural turmeric matrix employing polar—nonpolar sandwich (PNS) technology. The formulation was obtained by combining the hydrophobic and hydrophilic compounds—the three major curcuminoids, once extracted, are combined with turmeric water-soluble constituents, such as dietary fiber, carbohydrates, proteins, and lipophilic turmeric essential oil [84,98]. The synergism that occurs within turmeric matrix constituents enhances curcumin bioavailability thanks to physical stability improvement, protection from degradation, controlled release of curcuminoids, and, consequently, higher absorbability [84,99]. Human bioavailability was assessed by a pilot crossover study involving 12 healthy males and the formulation resulted in a 5.5-fold AUC increase compared to unformulated powder [83]. A single 500 mg oral dose of Cureit^®^ was also compared with the other two commercially available formulations, namely, a volatile oil and a phospholipid formula, in an open-label parallel-arm study. Forty-five healthy males were casually distributed to one of three groups and given 500 mg of the assigned formulation under fasting conditions. The results indicated that free curcumin AUC from Cureit^®^ was approximately 7.3-fold and 5.6-fold more effective, respectively, when compared with the volatile oil formula and phospholipid formula. Cureit^®^ supplementation was also shown to attenuate markers of muscle injury, decrease DOMS-associated pain symptoms, and lower CK blood levels. A randomized, placebo-controlled, double-blind trial was performed on 15 subjects, randomly assigned to the Cureit^®^ or placebo group. They underwent 4 days of intervention, during which they ingested a single oral dose of 500 mg of Cureit^®^ or placebo after a 45 min eccentric muscle injury test running downhill on a treadmill. Evidence showed a significant improvement after Cureit^®^ treatment against DOMS and a positive impact on muscle recovery [100].

Liposomes are part of a phospholipid-based vesicular system, among the most extensively investigated lipid drug carriers. Their success is due to their amphiphilic nature, high biocompatibility, and high affinity for biological membranes, since they can be rapidly taken up through pinocytosis or facilitated diffusion across lipophilic cell membranes [56,60]. Meriva^®^ [101] is a curcumin–phosphatidylcholine complex derived from soy lecithin, microcrystalline cellulose and 18–20% curcuminoids [56]. When nine volunteers, supplemented with a low or high dosage (209 mg and 376 mg of total curcuminoids, respectively) of Meriva^®^, as well as unformulated curcumin (1799 mg total curcuminoids), in a single oral administration, were randomly assigned one treatment for each 7-day washout period, free curcumin was not observed in any plasma samples but total curcumin appeared to be 18-fold higher in Meriva^®^ compared to the corresponding unformulated powder [60]. The administration of Meriva^®^ (500 mg/die, 4-month treatment) in association with glucosamine (500 mg) was found to alleviate the symptoms and decrease the need for further medications in patients affected by knee OA (*n* = 63) compared to the administration of chondroitin sulphate + glucosamine (400 mg + 415 mg; *n* = 61) [102]. Similarly, the administration of Algocur^®^ (containing 1 g of Meriva^®^) twice a day for 5 or 10 days significantly improved impaired physical function of male rugby players affected by osteo-muscular pain caused by traumatic injuries (*n* = 25). The effect was superimposable to conventional anti-inflammatory therapy (*n* = 25), but in the Meriva^®^ group, the adherence to therapy was greater due to the absence of adverse events [103]. The daily assumption of 500 mg of Meriva^®^ was also tested on visual acuity and optical coherence tomography (OCT) retinal thickness in patients with chronic diabetic macular edema. During a 3-month open-label study of 12 eyes, 92% showed a reduction of macula edema, 8% showed stabilization, and 0% showed a clinically meaningful increase [104]. Finally, a prospective phase II, single-center, single arm trial was conducted to investigate the efficacy and safety of curcumin as a complementary therapy to gemcitabine in advanced pancreatic cancer patients (*n* = 44). Meriva^®^ (2000 mg/day) was administered orally while gemcitabine was infused on days 1, 8, and 15 within a 28-day treatment cycle. Overall cycles were nine and Meriva^®^ was continued after treatment completion (up to 42 months). Results suggested preliminary evidence of the effectiveness of the co-treatment with Meriva^®^ and gemcitabine that could be compared to the combination of paclitaxel and gemcitabine with the advantage of producing less toxicity [105] (Table 2).

Unlike liposomes, solid lipid (nano)-particles (SLPs) are only suitable for hydrophobic drugs. They share with liposomes high biocompatibility, enhanced bioavailability, and high drug loading, but SLPs appear to be more stable and easier to manufacture [86,106]. Longvida^®^ is a patented formulation [107] manufactured by Verdure Sciences, USA, based on solid lipid curcumin particles (SLCPs), in which curcumin content is between 20% and 30% [56]. Pharmacokinetic studies were conducted on both healthy volunteers and osteosarcoma patients. Firstly, 11 subjects with diagnosed metastatic high-grade osteogenic sarcoma were divided into three groups and orally administered 2000 mg, 3000 mg, and 4000 mg of Longvida^®^ (curcumin content ranging from 400 mg to 1200 mg). Free curcumin concentration was improved and AUC values increased in a dose-related manner. Similarly, six healthy subjects were randomly clustered to receive a single oral dose of 650 mg of SLCPs or the same dose of unformulated curcumin. Free curcumin did not show any appreciable plasma level from the non-formulated curcuminoids extract, while SLCP AUC showed a clear increase in free curcumin concentration. The characteristic formulation of turmeric powder and phospholipid, the curcumin/lipid/antioxidant ratio, and globule size are indicated to underlie specific pharmacokinetic behavior and extended absorption of the formulation [85]. In Table 2 the discussed clinical studies are summarized.

## 3. Nanoformulations: What Is Known and What Is New

The lack of data regarding the protracted administration of high-dose curcumin formulations makes it difficult to foresee long-term effects on the human body. Conversely, epidemiological studies have revealed how the assumption of daily, low-dose, long-term curcumin preparations could produce significant health benefits on curry eaters [9,108]. Consequently, although dietary curcumin belongs to substances generally regarded as safe (GRAS), high doses could not be completely exempted by the onset of side effects [108]. This occurrence could be ascribable to the hormesis phenomenon, a dose–response relationship where low doses of xenobiotics induce stimulation, whereas high doses of the same substance cause inhibition or even toxicity. Among the strategies to enhance permeation and solubilization while providing low doses of drugs, nanoparticles represent one of the most attractive possibilities, particularly for all substances belonging to class IV in the biopharmaceutics classification system (BCS), such as curcumin. Nanoformulations provide substantial benefits due to their fine features that make them extremely versatile. Indeed, because of their tiny size (1–100 nm), they can easily penetrate through the intestinal mucus layer via transcytosis, increasing nutraceutical bioavailability [109]. Moreover, the small dimension prevents nanoparticles from aggregating and settling since they are affected by the brownian motion that overcomes the force of gravity and keeps the particles moving randomly. Furthermore, the reduction of particle size makes their surface area become larger so that any chemical reactions occurring at their surface will be faster. Eventually, the chemical, physical, optical, electronic, and magnetic properties of materials could differ enormously when referred to the nanoscale, giving nanoparticles unique properties [109]. All the features listed above match together the major advantages and disadvantages of nanoparticles since their behavior is often different, or even unpredictable, from the same material in bulk.

As a result of the growing interest in environmental issues from both consumers and producers, another point in favor of nanomaterials is their green synthesis methods that take advantage of green chemistry [109,110]. Two main approaches are reported top-down and bottom-up. While the top-down process consists of breaking down larger particles into small ones using intense mechanical forces, in the bottom-up approach the molecules associate with each other, thanks to the creation of appropriate conditions favoring intra-molecular attraction [109].

Nanotechnology is not new to the food industry and, though still portrayed as dangerous for health concerns, its applications are numerous: from agricultural production, protecting crops, or feeding plants, to enhancing and preserving food quality from the processing to the packaging [109]. Nevertheless, encapsulation technology to control and target the delivery and bioavailability enhancement of healthy nutrients and nutraceuticals remains the most attractive aspect of nanomaterials.

### 3.1. Nanotech in Bioavailability-Boosting Systems

Colloidal delivery systems and excipient foods are among the latest approaches developed to enhance bioavailability of nutraceuticals like curcumin. On one hand, excipient foods do not have any bioactivity themselves but, once in the gut, form an environment that increases bioaccessibility, stability, or absorption of any substances co-ingested with them [106,109,111]. In vitro studies tested the effects of excipient emulsions on stability and bioaccessibility of powdered curcumin at both 30 °C and 100 °C, to simulate conditions commonly found in foods such as dressings or cooked sauces. Excipient emulsion was prepared by mixing corn oil (10% *w*/*w*) and Tween-80 aqueous solution (90% *w*/*w*) with a high-shear mixer and the use of a microfluidizer allowed researchers to obtain particles with different diameter sizes—large, medium, or small droplets, respectively. Powdered curcumin was dissolved in the excipient emulsion at a percentage of 3% and the resulting mixtures were then incubated at either 30 °C for 30 min or 100 °C for 10 min. From the analysis of the collected samples, it emerged that no appreciable changes were found in curcumin excipient emulsion mixtures—in droplet characteristics, electrical charge, or droplet microstructures of the samples incubated at 30 °C for 30 min—while a small increase in the mean droplet diameters and the appearance of some larger oil droplets in the systems that firstly contained small droplets occurred after heating at 100 °C for 10 min, indicating that some aggregation and coalescence happened during incubation at high temperatures [112,113]. As for curcumin bioaccessibility, the studies demonstrated that a significantly greater amount of curcumin was found in the emulsion after incubation at 100 °C than 30 °C and it was considerably higher for large and medium droplets than for the small droplets at 30 °C. Moreover, some curcumin crystals were clearly observed in all the mixtures incubated at lower temperatures, suggesting incomplete dissolution. Since the excipient emulsion incubated at 100 °C contained a major amount of curcumin, it was tested for its potential biological fate passing through a three-step GI tract model. This evidence supported the involvement of droplet sizes of the initial excipient emulsion on the curcumin concentration in digesta or mixed micelle phases produced from lipid digestion (large > small ≈ medium), however curcumin bioaccessibility did not result from droplet size, since the same amount of mixed micelles formed for all three droplet sizes studied [113]. Excipient emulsions of curcumin, when compared with curcumin dissolved in corn oil or buffer solution (either at 30 °C for 30 min or 100 °C for 10 min) and exposed to simulated GI tract conditions, revealed a higher curcumin concentration in the mixed micelle phase, highlighting the impact of the nature of the food matrix on the digestion fate [112].

Conversely, colloidal delivery systems enable the encapsulation of low-bioavailable bioactive agents inside nanoparticles. The latter are specifically designed for specific nutraceuticals to be encapsulated in an appropriate food matrix and incorporated into and tailored corresponding to the needed characteristics in the final product [106]. The systematic approach to obtain the most appropriate final-purpose delivery system is the recently developed delivery by design (DbD) principle. It consists of a seven-stage process that aims to design, fabricate, and test colloidal delivery systems suitable for commercial applications. All stages are summarized in Table 3. Briefly, stages 1 and 2 involve defining the physicochemical properties of either the active agent or the final product, highlighting the major obstacles to overcome and the functional attributes, respectively. Therefore, stage 3 entails defining the characteristics that a colloidal delivery system should possess to effectively encapsulate the active ingredient. Once the colloidal delivery system has been defined, it is necessary to qualify the features of the colloidal particles and select the most suitable delivery system, bearing in mind the provided features (stage 4). Stages 5 to 7 involve the optimization of the fabrication method: (i) definition of the manufacturing approaches to ensure that the production is economically reliable for commercial applications, (ii) establishment of testing protocols for evaluating and ensuring the performances of a delivery system in a specific end-product, (iii) monitoring and recording properties of delivery systems and end-products to make it easier to adjust, where needed, the factors that can affect their properties [54,106].

The DbD approach has been tested on curcumin as a case study. In the reported case study, since curcumin is a water-insoluble, low-molecular-weight, and crystalline constituent that cannot be integrated into an aqueous-based functional beverage without being dispersed in colloidal particles, it has been encapsulated to design a fortified nutritional drink like the Ayurvedic “golden milk”. As this beverage appears cloudy, yellowish, and creamy, oil-in-water nanoemulsion was selected as the most appropriate delivery system to reach the goal [54,106].

The range of functional food matrix where curcumin could be incorporated is extremely wide and each food type has its own characteristics (bread, beverages, sauces, dressings, frozen meals, cereal bars), therefore, colloidal delivery systems should fulfill each requirement according to the end-product’s variability. An overview of the major food-grade colloidal delivery systems that efficiently encapsulate curcumin is given below. Moreover, the latter, categorized as simple, delivery systems could be used as building blocks to prepare complex delivery systems through structural design techniques, such as embedding, clustering, coating, or mixing [54].

#### Lipid-Based Colloidal Delivery Systems

Micelles, emulsions, nano- and microemulsions, liposomes, and solid lipid nanoparticles (SLNPs) or nanostructured lipid carriers belong to this category. As the lipid-based colloidal particles are surely the most widely investigated and used delivery system for curcumin, detailed information from clinical studies involving these carriers have already been given in the previous section and the current paragraph would only deepen their specific use according to the DbD practice.

Micelles are among the smallest colloidal particles used to deliver drugs, since their diameter typically ranges from 5 to 20 nm. Consequently, they hinder the light scatter and appear optically clear, which makes them appropriate for application in beverage products that are supposed to be transparent. Micelles are thermodynamically stable systems formed from natural or, more often, synthetic surfactant molecules that self-assemble when the critical micellar concentration (CMC) is reached. They organize in particles with a hydrophobic core, containing surfactant tails and a hydrophilic shell made up of the surfactant heads. Their fabrication is somewhat simple and consists of heating and/or mixing the surfactant, the bioactive agent, and water together. The most used food-grade surfactants for micelles production include Tween-20 and Tween-80 [81], although micelles could also be formed from natural biosurfactants, such as sophorolipids, synthesized by yeast fermentation. Among their advantages, it is important to consider the high stability and good bioavailability. However, sophorolipids need a high concentration of surfactant to self-assemble, which represents an obstacle in terms of costs and consumer acceptance [54,86,106,111]. Microemulsions and micelles are structurally and functionally similar, except for the presence of some oil in microemulsions that form an additional core located between the surfactant tails, which is responsible for their larger dimensions. Capryol-90, Transcutol P, Cremophor-RH40, soybean oil, soy lecithin, and Tween-80 are some of the food-grade ingredients used to prepare microemulsions [106].

Emulsions and nanoemulsions are usually formed by two immiscible liquids (oil and water) that are stabilized by emulsifiers, thickening, jelly agents, or many other food-grade ingredients. They differ mainly for their dimensions since their diameters typically range from 10 to 100 nm in the case of nanoemulsion or from 100 nm to 100 µm for emulsions. This difference leads to different physicochemical properties and functional attributes—while emulsions appear cloudy or opaque, nanoemulsions may appear clear. Considering that both are thermodynamically unstable, it is necessary to apply mechanical or chemical energy to produce them—high-pressure homogenization, microfluidization, and sonication are processes generally employed by the food industry and are relatively inexpensive [114]. Curcumin-loaded emulsion could be prepared from proteins or polysaccharides, such as soy carbohydrates or casein, whey protein, Arabic gum, lecithin, or Tween-80 [115,116]. Unfortunately, the instability affects their storage as they tend to separate and break down [54,86,106,111]. After comparing curcumin oil-in-water nanoemulsion and emulsion, it emerged that curcumin stability in conventional emulsion was slightly higher, but nanoemulsions had greater stability [117]. However, a curcumin nanoemulsion prepared from organogel in the oil phase with Tween-20 as an emulsifier showed a ninefold increase in curcumin oral bioavailability when compared with unformulated curcumin [118].

Liposomes are self-assembled systems formed by one or more phospholipid bilayers. Their dimensions vary considerably according to the formulation or the fabrication method. Commonly, particles with a diameter smaller than 100 nm are called nanoliposomes, while particles whose diameters are larger than 100 nm are the so-called liposomes. There are a variety of methods for fabricating liposomes, many of which use organic solvents that are inacceptable for food applications; the pH-driven procedure is an organic solvent-free technique based on the deprotonation and dissolution of hydrophobic drugs under alkaline conditions followed by neutralization with an acidic solution of surfactants. Lowering the pH by mixing the solutions forces the hydrophobic molecules of the drug to precipitate and segregate inside the surfactant molecules [119]. According to the desired end-product, liposomes could be designed to have different sizes and, consequently, different appearances, which means they can be integrated in several marketed products. Moreover, liposomal delivery systems could be fabricated from all natural ingredients to meet the compliance of consumers, such as egg lecithin [120] or, more commonly, soy lecithin, milk, rapeseed, canola seed, cottonseed, and sunflower [115]. Despite these benefits, industrial manufacturing is quite expensive, and they are not robust enough to withstand the environmental stresses encountered in foods [54,86,106,111].

SLNPs are comparable to oil-in-water emulsion, except for the lipid phase NPs, which are solid rather than liquid. Indeed, they could be prepared by homogenization of a melted fat with an aqueous emulsifier solution at a temperature higher than the fat melting point, the cooling of the emulsion below the crystallization temperature of the fat leads to the solidification of the fat droplets. SNLPs are particularly suitable for loading hydrophobic drugs that should be locked into the solid interior during the cooling step. As for the other colloidal particles, particle size could range from 20 to 1000 nm, giving the suspension of transparent or cloudy appearances. Food-grade ingredients to synthesize SLNPs include propylene glycol, glycerol stearate, esters of fatty acids, palmitic acid, soy lecithin, and glyceryl behenate in the lipid phase and Tween-80 solutions in the aqueous phase [121,122]. Unfortunately, if not properly formulated, SLNPs tend to expel the bioactive agent and to form particle aggregates. Apart from this, their fabrication profits by common and green processing operations, such as high-pressure homogenization, and can be manufactured on a commercial scale with food-grade ingredients. The overcoming of fabrication issues could be achieved by developing lipid particles that cannot form highly ordered crystalline structures, thanks to the use of a mixture of fat with diverse melting points or lipids that remain in an amorphous phase when cooled [86,106].

### 3.2. Biopolymer-Based Nanoparticles

The most common food-grade biopolymers used to fabricate colloidal delivery systems are either synthetic or natural [106]. When dispersed in a solvent, the biopolymer-based particles form a 3D network capable of trapping both the active agent and the solvent inside. Biopolymer nanoparticles (*d* < 100 nm) could be prepared from a variety of methods, among which, the simplest consists of a dispersion of drug-loaded polymeric particles in organic solutions that precipitate in aqueous media. Ultrasonication and microwaves are the latest green methods developed. Fabrication method affects the characteristics of the biopolymers, which allows for the designing of particles with specific delivery properties, such as retention or release of the active agent inside, especially for those preparations where a triggered or sustained release is required [106,111]. Poly-(lactic-co-glycolic acid) (PLGA) is a biodegradable aliphatic polyester commonly utilized for fabricating curcumin nanoparticles [123,124,125]. Several studies have underlined that PLGA has high encapsulation efficiency—curcumin loading is high and the nanoformulation offers great stability and curcumin cellular uptake is enhanced and exhibits pro-apoptosis and anti-proliferative effects on the growth of metastatic cancer (MDA-MB-231 and A2780CP) cells compared to free curcumin, while showing no effects on cell viability of the polymer itself [123]. Moreover, the kinetics of curcumin release display an initial burst release with about 43 ± 3% of the drug released from PLGA within the first hour, followed by a negligible amount of curcumin (>5%) released between 1 h and 24 h. As a matter of fact, this release/retention characteristic is a needed feature for oral administration of BCS class II substances, such as curcumin, as the improvement of the dissolution ratio could increase the bioavailability that is inhibited by their scarce solubility [124]. Despite the fact that PLGA is one of the most extensively studied biopolymer-based nanocarriers for curcumin, polysaccharides and proteins are perceived as healthier by consumers when compared not only to synthetic colloidal particles, but also to the most common lipid-based ones [106]. Many studies have focused on encapsulating curcumin with chitosan nanoparticles and the results generally revealed bioavailability and solubility enhancement in encapsulated curcumin compared to native powder [126,127,128,129]. When orally administered to rats (10 or 50 mg/kg), chitosan–curcumin nanoparticles showed an 11.45-fold increase in bioavailability compared to native curcumin and persistence in blood circulation up to 7 days, probably due to the bioadhesion properties of the polymer itself to the intestinal mucosa [129]. In another study, chitosan–curcumin nanoparticles synthesized using an ionotropic gelation technique indicated an initial burst release of curcumin for 2 h, further followed by a sustained release of the drug up to 96 h. Moreover, the formulation showed good stability at storage temperature for two months (4 °C or 25 °C). When the cytotoxic effect was evaluated, a relatively low concentration was found to be successful at inhibiting HeLa cell proliferation, while the chitosan nanoparticles alone demonstrated no considerable decrease in cell viability and satisfactory biocompatibility [126]. Caseins represent a valuable natural alternative to polysaccharides to encapsulate hydrophobic drugs. Indeed, beta-caseins from camel milk were found to form an efficient self-assembling nanostructured carrier for curcumin. The presence of the micellar structure increases curcumin solubility, bioavailability, and antioxidant activity, while proving a cytotoxic effect on human leukemia cells line K-562 [130]. Interestingly, curcumin microparticles, prepared from whey protein isolated via spray drying method, have been integrated into a model food matrix (yogurt) to test whether the characteristics of retention or release, as well as bioaccessibility or sensory properties, were affected. Findings revealed that even if the presence of yogurt affects the release of curcumin from the complex, the kinetic features, such as burst or prolonged release, are remain, highlighting the role of food components in the bioavailability of nutrients. On the other hand, curcumin microparticles did not affect yogurt properties, except for color and curcumin flavor [131]. It is worth mentioning that curcumin-loaded Pluronic^®^ polymeric micelles gained attention as a novel drug delivery strategy through complex microbial communities into target cells. Indeed, Pluronics^®^ are copolymers of polyethylene oxides (PEO) and lipophilic polypropylene oxide (PPO), organized in a triblock structure (PEO-PPO-PEO), characterized by an amphiphilic nature that enables self-aggregation in aqueous solution. Although further investigation is needed for confirming, improving, and better understanding the early results, curcumin-loaded Pluronic^®^ F-127 micelles showed good chemical stability within 30 days from the fabrication and encouraging results indicate this product as an alternative therapy to inhibit biofilm proliferation processes in oral cavity diseases, such as dental caries [132].

### 3.3. Naturally Derived Colloidal Particles

In trying to find more natural and healthier alternatives to processed food, it was necessary to investigate the possibility of employing preexisting natural colloidal particles, such as fat globules found in bovine milk or oil bodies in plant-based milks. For instance, curcumin has been successfully encapsulated inside milk fat globules in bovine milk and oil bodies in soybean milk. In both experiments, curcumin was loaded in the oily globules thanks to the PH-shift method without any alteration of the beverages. It also showed good stability under storage in refrigerated conditions, particularly between 5 and 20 °C. Bioavailability was assessed using simulated GI tract conditions and was around 40% for bovine milk fat particles, while 55–59% was available for absorption in the mixed micelle phase from soybean milk [133].

### 3.4. Nanotech in Targeted Delivery Systems

The drug-targeting approach is an integral component of the overall process of drug development. Indeed, targeting the drug selectively to the cells where the pharmacological action is needed increases the therapeutic efficiency while decreasing undesirable side effects [134,135]. The conjugation of the drug-targeting approach with a nanodelivery systems would allow the nutraceuticals to be at the proper place at the correct time, increasing the beneficial effects, decreasing undesirable toxic effects, limiting nutraceuticals metabolism, and boosting their bioavailability. As many strategies have been taken under consideration by scientists, three of them are discussed below.

#### 3.4.1. Peyer’s Patches

Peryer’s patches (PPs) are lymphoid nodules that aggregate in follicular structures that cover the ileum portion of the small intestine, giving birth to the gut-associated lymphoid tissue (GALT). PP follicles consist of a core containing B lymphocytes, dendritic cells, and macrophages that are detached from the intestinal lumen by a specific layer, the follicle-associated epithelium (FAE), which incorporates enterocytes, goblet cells, and modified epithelial cells termed microfold cells (M cells) [136,137]. M cells are specialized enterocytes whose role is to bind and transfer xenobiotics from the lumen to the underlying immune cells, which then ignore or trigger the immune response depending on the antigen being processed [137]. Although several studies have already taken PPs into consideration for drug-targeting purposes [138,139,140], most of the orally administered drugs still get metabolized before reaching the systemic circulation, unless selective uptake occurs in lymph. Lipid-based and polymeric nanoparticles are the delivery systems known to pass the intestinal membrane and enter the lymphatic circulation, the first being absorbed via chylomicrons/enterocyte uptake, while the second are taken up by M cells [136], indeed, they can transport substances across the intestinal wall through endocytosis, pinocytosis, micropinocytosis, and phagocytosis and deliver it to the basolateral side by exocytosis. Accordingly, M cells represent an intriguing opportunity to target drugs at PPs, though the uptake strongly depends on the particle size, zeta potential, and surface modifications of the particulate system. In consequence, non-ionized, hydrophobic nanoparticles, sized below 1 μm (more precisely below 200 nm) are easily transcytosed by M cells. Furthermore, surface functionalization with specific M cells ligands, such as lecithin, IgA, or antibodies, enables more specific targeting [136,137]. Despite these characteristics, the main issue with M cells uptake remains the integrity of the substance reaching the intestinal wall, thanks to carriers specifically tailored for the purpose.

As already described above, polymeric nanoparticles trap hydrophobic bioactive agents inside a 3D network structure, preventing enzymatic degradation occurring over the GI tract and delivering the intact drug with unchanged PP levels [137,138]. Size, shape, surface properties, and surface charge deeply influence the rate and length of PP absorption. Indeed, the transport rate of hydrophobic particles, whose dimensions are 50–200 nm, rod-shaped, and negatively charged is higher than bigger, sphere- or disc-shaped hydrophilic particles with neutral or positive surface charge. Nevertheless, an in vivo study proposed the encapsulation of curcumin in the lauroyl sulphated chitosan (LSCS-CUR), a hydrophilic and positively charged polymer. LSCS-CUR nanoparticles displayed better aqueous solubility than unformulated curcumin and better mucoadhesion than chitosan alone, which plays a pivotal role in enhancing curcumin cellular uptake by expanding the absorption surface. Similarly, mucoadhesion is involved in the longer persistence of curcumin in blood up to 7 days after oral administration. It is noteworthy that tissue distribution analysis showed a higher concentration of curcumin localized in the intestinal portion, particularly at the duodenum level [129]. As mentioned above, the rate of transport or release of bioactive substances reflects the nature of the polymer matrix. In fact, encapsulating curcumin in PLGA nanoparticles allows biphasic release of the active ingredient—a small amount is instantly issued from the formulation, followed by a slow and steady release of the remaining. This release trend has further been demonstrated in an animal test, where Wistar rats were fed with curcumin encapsulated in PLGA nanoparticles or curcumin alone. The formulation showed a 24 h steady release, as opposed to unformulated curcumin, which achieved the maximum plasma concentration after 30 min from oral intake and then fell following its metabolism [141].

Dendrimers are nanometer-sized treelike structures characterized by an initiator core of monomeric units covered by an outermost exterior layer that provides a multifunctional surface for surface chemistry modification [86]. Once again, dendrimer size, charge, and concentration influence their absorption rate at intestinal level, which can occur for molecules with diameters up to 3 nm, either through the paracellular or transcellular routes. It is noteworthy that dendrimers appeared to express specific tropism for the small rather than the large intestine. Although only few studies have been conducted on curcumin–dendrimer formulation, there is evidence of improved aqueous solubility and bioavailability of dendrosomal curcumin [142,143], cytotoxic activity and tumor target efficacy against several human cancer cell lines [142,143,144,145], reduced cancer cell proliferation, and apoptosis induction with a dose-dependent cytotoxicity showed by polyamidoamine curcumin dendrimer formulation [146].

Self-microemulsifying drug delivery systems (SMEDDSs) are dry microemulsions without an aqueous phase; they can be inserted into capsules or converted into solid dosage forms, such as tablets or pellets, that, upon oral administration, are dispersed in the gut lumen, spontaneously forming a microemulsion [86]. This fact allows not only the active agent to be presented in a solution form in the gut, but also, since SMEDDSs do not need biliary salts and enzymes to be digested, limits possible absorption alterations due to individual variability. Lastly, enhancing drug solubilization and improving lymphatic target efficiency of SMEDDSs increases membrane permeability in the GI tract and lymphatic uptake of drugs, thus lowering the drug amount required to have a clinical effect and reducing the systemic side effects, thereby improving patient compliance and cost of therapy [137]. Several studies conducted on animals have agreed, finding a meaningful increase in absorption and bioavailability when curcumin SMEDDS formulation is compared to unformulated curcumin [147,148,149,150,151].

#### 3.4.2. Inorganic Nanoparticles

Metallic or non-metallic particles, sized between 1 and 100 nm are commonly referred to as inorganic nanomaterials [110]. As for other nanoparticles, their tiny features, small size, large surface area, and higher reactivity mark the major benefits and the deeper concerns associated with their use [109], since the average particle diameter, size distribution, and charge affect the physical stability and the in vivo distribution of nanoparticles. Nevertheless, inorganic nanoparticles offer a wide range of applications in the biomedical field as they are already used in diagnostic imaging, gene vehicles, protein separation and purification, cellular tracking, and more [110,152]. Interestingly, many of these inorganic nanomaterials are routinely used in food and agricultural fields, as pesticides to treat the crops or as additives to provide food and beverages with specific optical characteristics [109]. Inorganic nanoparticles provide high payload loading and payload protection, tunable degradation rates, localized target delivery, enhanced permeation, or accumulation into specific tissues due to the base material properties and chemistry surface functionalization chosen—all these characteristics make them an extremely practical targeted drug delivery system for nutraceuticals.

Among metallic nanoparticles, gold and silver (Au and Ag, respectively) particles have garnered attention because of their high plasma absorption, biocompatibility, versatility, and antimicrobial properties [153]. Gold nanoparticles are mainly synthesized by chemical methods that encompass the reduction in gold salt with the help of reducing agents, although plant-based materials, sunlight irradiation, and green chemistry have been used as green methods. The modulation of thiol/gold ratio during the fabrication process permits control of the size and shape of nanoparticles, which are closely related to nanoparticle distribution and toxicity [152,153]. In a hypertrophy-induced animal model, encapsulating curcumin in gold-loaded PLGA nanoparticles (CAu-PLGA NPs) resulted in the inhibition of cardiac hypertrophy due to the structural modification of curcumin in CAu-PLGA NPs that increased its bioavailability, solubility, and absorption, improved blood circulation and resistance to enzyme degradation; furthermore, CAu-PLGA NPs allow a more controlled and complete drug delivery, increasing therapeutic efficiency [154]. Another in vivo study assessed the potential and specificity of curcumin-encapsulated chitosan-graft-poly (*N*-vinyl caprolactam) nanoparticles containing gold nanoparticles (Au–curcumin–TRC NPs) in targeted cancer drug delivery. Interestingly, Au–curcumin–TRC NPs were found to facilitate extended persistency of curcumin in the blood for up to a week, compared to curcumin alone, which disappeared from circulation after 6 h, and maximum curcumin accumulation was observed in the tumor compared to other organs [155]. To support these findings, several in vitro studies proved cytotoxicity and increased uptake of curcumin-functionalized gold nanoparticles (curcumin–Au NPs) in human prostate cancer cell culture [156]. Moreover, antiproliferative and apoptotic effects on colon and breast human cancer cell lines [157] and a synergistic effect produced by both the gold core and curcumin that triggers the generation of ROS, which consequently initiates the apoptotic pathway with the release of protein Bax, activation of PARP cleavage, and DNA fragmentation, were observed [158].

Silver nanoparticle synthesis takes advantage of chemical, physical, or green methods based on fungi, bacteria, algae, and plants [153]. As with other nanoparticles, shape, size, concentration, colloidal state, and surface charge influence the antimicrobial properties exhibited by silver nanoparticles that lead to the fabrication of curcumin-loaded silver nanoparticles as antibacterial agents. Curcumin–Ag NPs of 25–35 nm were synthesized and demonstrated efficacy against Gram-positive and Gram-negative bacteria, lowering the minimum effective curcumin concentration fourfold (20–5 mg/L) and toxicity to human keratinocytes. Moreover, skin biocompatibility studies showed an induced anti-inflammatory effect on human macrophages [159]. Although daily approximate silver intake by humans through food and/or water is 0.4–30 μg, which indicates that in the given range its consumption is safe, there are some reports where silver nanoparticle-related toxic effects have been observed in animal models [153] that need further investigation.

Magnetic nanoparticles (MNPs) consist of a metallic oxide core or metal that could be functionalized within an inorganic metal or polymer coating. Among MNPs, magnetic iron oxide nanoparticles, otherwise known as magnetite or Fe_3_O_4_, have been approved by US Food and Drug Administration (FDA) for clinical imaging and drug delivery applications as they are considered safe and biocompatible [153]. Among the variety of existing fabrication methods, green biosynthesis is usually selected over other techniques due to higher biocompatibility, biodegradability, and non-toxic surface coating of green materials [153]. Curcumin-loaded MNPs were evaluated both in vitro and in vivo in pancreatic cancer for assessing therapeutic efficacy. The MNPs–curcumin formulation appeared efficiently dose-dependently internalized by human pancreatic cancer cells, whereas the in vivo study showed the ability of the MNPs–curcumin formulation to suppress pancreatic tumor growth. Furthermore, the MNPs–curcumin formulation improved serum bioavailability of curcumin in mice up to 2.5-fold compared with free curcumin, without any sign of hemotoxicity [160]. Another in vivo study tested the hemocompatibility and bioavailability of polyethyleneimine and glutathione functionalized iron oxide nanoparticles for targeted curcumin, resulting in up to 2.5-fold enhanced serum bioavailability for curcumin-loaded MNPs compared with free curcumin [161].

Mesoporous silica nanoparticles encountered the favor of the biomedical industry thanks to their high chemical inactivity, biocompatibility, thermal stability, high hydrophilicity, high loading capacity, and resistance to microbial attack. As many synthesis methods are employed for fabrication, the green process of silica nanoparticles fabrication is gaining more and more resonance since it relies on the use of agricultural waste. Amorphous silica, to which mesoporous silica belongs, was considered secure by the FDA at an oral intake of up to 1500 mg/die for foods and as a delivery vehicle [162,163], contrary to its crystalline form, involved in emphysema, pulmonary tuberculosis, lung cancer, and fibrotic lung disease (silicosis) [153]. Curcumin-loaded mesoporous silica nanoparticles were prepared and coated with polyethylene glycol (PEG) and ultimately conjugated with the targeting moiety transferrin (Tf) for targeting pancreatic cancer cells. Data showed enhanced uptake and, consequently, higher cell killing in vitro, while in vivo results on animal models clearly demonstrated inhibition of tumor growth and prevention from distant metastasis spreading [164].

### 3.5. Microbiota

It is largely acknowledged that the gut microbiota is pivotal for maintaining human health and conditions recognized as dysbiosis are correlated with the onset of chronic pathologies, including allergies, autoimmunity, GI disorders, metabolic and cardiovascular disorders, cancer, and CNS dysfunctions [165,166]. Gut microbiota composition tightly depends on host genetics and interpersonal variance and it is profoundly influenced by age, diet, lifestyle, and environmental factors [166], so that any improper diets could determine alterations in the gut microbiota composition, leading to intestinal permeability changes and low-grade gut inflammation [167].

Recently, a growing number of studies have focused on the correlation between curcumin and gut microbiota after oral administration as a high concentration of polyphenols is found in the GI tract [168,169,170]. Taken together, these results highlight a bidirectional interaction [166,167]: on one hand gut microbiota participate actively to curcumin metabolism, as it acts as a metabolic bioreactor, producing various enzymes that contribute to curcumin biotransformation in metabolites, which display similar or superior activity compared to curcumin itself [171,172,173]; in contrast, the administration of curcumin promotes the growth of beneficial bacterial strains, inducing a shift in the ratio between beneficial and pathogenic species [168], improving intestinal barrier functions [174,175] and counteracts the expression of biomarkers of inflammation and oxidative stress [169].

Though these studies were executed under different conditions, using various doses or formulations, thus being hardly comparable, they give the path to novel microbiota-targeting curcumin-based therapies.

### 3.6. A Brief Overview of Fabrication Issues

In order to deepen commercial pros and cons of nanoformulations used as strategies to improve drug bioavailability, it is firstly necessary to establish their intended use. Ingredients, fabrication processes, and research and development investments would vary considerably depending on food and food-related production or dietary supplement production, inevitably affecting the cost to both manufacturers and consumers.

When referring to the food industry, it is important to consider, next to the ease of fabrication and related costs, consumer acceptance, which influences consumer choice. All the lipid-based colloidal delivery systems, as well as the naturally derived biopolymers, could be produced from natural food-grade ingredients commonly perceived as healthier by consumers, although only emulsions, nanoemulsions, and SLNPs could be fabricated in a large-scale process, using common and inexpensive processing operations that are already used in the food industry, like high-pressure homogenization, microfluidization, or sonication [106,176]. According to curcumin bioavailability enhancements, data suggest that nanoemulsions are the most suitable delivery system since they could be fabricated from a variety of cheap, plant-based emulsifiers (soy, pea, lentil, bean proteins) and lipids (corn, sunflower or coconut oil) on a commercial scale [106,177]; if properly formulated, they have relatively good stability over the stresses encountered by foods during production and storage [178], as well as high bioavailability [179]. Moreover, they are extremely versatile since they could be incorporated into a beverage or dried into a powdered form [180].

On the side of clinical or pharmaceutical applications, the latest approaches investigated above, such as inorganic nanoparticles, nanopolymers, or dendrimers, suffer from a deficiency of data in terms of human pharmacokinetics and pharmacodynamics, which limit predictable evaluation of costs related to raw materials. Nevertheless, fabrication methods have been well investigated. Concerning polymer nanoparticles, among many production approaches [181,182,183], nanoprecipitation (emulsion–solvent evaporation method) and the emulsions–diffusion method are the easiest to scale up [183] and the simplest to perform [86], although green methods based on ultrasonication and microwaves are emerging in order to reduce toxic reagents and simplify the procedure to allow economic scale-up [86,182].

Inorganic nanoparticles could be fabricated using numerous methods, including chemical, physical, and bio-based green methods (sunlight irradiation, from bacteria, fungi, yeast, algae, and plants) [110]. As already mentioned above, top-down and bottom-up approaches are among the easiest. Due to the fact that inorganic nanoparticles are already used in diagnostic medicine and imaging, scaling up their production is a reality, although not free from costs.

As they consist of a repetition of monomeric interior units encircled by the outermost region, dendrimers are generally synthesized by a sequential repeat of reaction steps. Indeed, they are formed of a core, from which a number of spacers depart, whose ends combine with a high degree of branching that allows surface chemistry functionalization with proteins, amino acids, fatty acids, and polymers in order to enhance solubility and reduce toxicity [184,185]. The use of dendrimers as drug carriers is still the subject of study, though recent applications include nanomedicines, gene-delivery vectors, biological adhesives, and imaging agents [137,185,186,187,188]. Finally, SMEDDSs belong to the emulsion class since they are dry microemulsions without an aqueous phase and they have the great advantage to spontaneously form emulsions in vivo without any energy input [86,137]. According to their high targeting efficiency, it is possible to reduce the drug amount required to have a clinical effect, thereby improving patient compliance and the cost of therapy [137].

Taking these evaluations into account and considering the absence of data regarding some aspects of the newest drug delivery approaches, it is appropriate to affirm that nanoemulsions are, at present, the most suitable, well-investigated, and low-cost approach to enhance bioavailability of curcumin, either as a drug or a dietary supplement to enrich food and beverages.

## 4. Conclusions

Currently, the polyphenolic compound curcumin is one of the most investigated natural products and its beneficial effects in several human disorders have been established, along with a low toxic profile. During the years, numerous curcumin drug targets have been described, providing the molecular basis for the pharmacological action. Unfortunately, the bioavailability and delivery of curcumin are the main obstacles for its effectiveness, precluding the use in medicine. In this review, we have explored the most promising and modern strategies for improving curcumin bioavailability, offering a comprehensive overview regarding this field. Surely, the progress in the nanoparticle field could provide, in the near future, further curcumin-based complexes with significant pharmacological profiles that can be developed as effective therapeutic agents for treating human diseases. The advancement in this field holds great promise to employ this polyphenol in medicine. Moreover, due to the multifunctional profile of curcumin, an efficacious delivery method may offer a better possibility of effective prophylaxis or treatment in complicated multifactorial disorders, such as systemic inflammatory diseases and cancer, supporting the curcumin-based agents as preventive and disease-modifying therapeutics.

## Figures and Tables

**Table 1:** The pharmacokinetic profile of curcumin considering various commercially available, curcumin-based formulations.

Formulation	Commercial Product	Study Design	Subjects	Sample Hydrolysis	Food Administration	Intervention	Dose	AUC_(0−t)_ (ng*h/mL)	Refs.
Curcumin + piperine	Curcumin C^3^ Complex^®^ + Bioperine^®^	Randomized crossover	8 (10) Asian males 20–26 yo	No	Overnight fasting administration with 150 mL of water and standard meals	Formulation Control	2000 mg curcumin + 20 mg piperine 2000 mg curcumin	80 ± 10 ^a,2^ 4 ^a,2^	[69]
Curcumin + piperine	Curcumin C^3^ Complex^®^ + Bioperine^®^	Randomized crossover	6 males	Yes	-	Formulation Control	2000 mg curcumin + 5 mg piperine 2000 mg curcumin	15.55 ^d,1^ 8.44 ^d,1^	[42]
Curcumin + piperine	Curcumin C^3^ Complex^®^ + Bioperine^®^	Open-label, uncontrolled phase I pilot study	10 European males 23–33 yo	No	Overnight fasting	Formulation	12,000 mg curcumin + 5 mg piperine	ND ^6^	[72]
Curcuminoids + turmeric essential oil	BCM-95^®^CG Biocurcumax™	Pilot study crossover	11 (Gender not reported) 28–50 yo	No	Overnight fasting	Formulation Control	2000 mg curcuminoids 2000 mg curcuminoids	3201.28 (μg*h/g) ^b,7^ 461.86 ^b,7^	[77]
Micellar curcumin	Novasol^®^	Single-blind crossover	23 13 females 19–26 yo 10 males 20–28 yo	Yes	12 h overnight fasting; drugs mixed with 50 g woodruff syrup; meals with neither food nor water restriction	Formulation Control	410 mg curcumin 410 mg curcumin	4474.97 ± 1675.02 ^c,4^ 24.16 ± 42.58 ^c,4^	[62]
Micellar curcumin	Novasol^®^	Single-blind crossover	23 6 females 23 ± 2 yo 5 females 67 ± 3 yo 6 males 26 ± 2 yo 6 males 62 ± 3 yo	Yes	12 h fasting administration with water	Formulation Control	80 mg curcumin 80 mg curcumin	212.48 ± 76.36 ^c,5^ 2.54 ± 4.20 ^c,5^	[81]
Curcumin + cyclodextrin	Cavacurmin^®^	Randomized double-blind crossover	12 11 males 1 female 23.0 ± 2.4 yo 1 African -American and 11 Caucasians	Yes	10 h overnight fast; at 4 and 8 h after administration curcumin-free meals were served (first mealtime: 40 g chocolate whey protein isolate and 80 g instant oatmeal dissolved in 30 mL of water + 473 mL of water to drink; second mealtime: 230 g turkey breast, 2 slices of whole wheat bread, 15 g light miracle whip, 170 g of fat free Greek yogurt, and 473 mL of water)	Formulation Control	376 mg curcuminoids 1800 mg total curcuminoids	327.7 ± 58.1 ^a,4^ 3.9 ± 0.5 ^a,4^	[57]
Curcumin + hydrophilic carrier	Curcuwin^®^	Randomized double-blind crossover	12 11 males 1 female 23.0 ± 2.4 yo 1 African-American and 11 Caucasians	Yes		Formulation Control	376 mg curcuminoids 1800 mg total curcuminoids	307.6 ± 44.6 ^a,4^ 10.8 ± 1.7 ^a,4^	[58]
Curcuminoids + fenugreek dietary fibers	CurQfen^®^	Randomized double-blind crossover	50 43 males 7 females 24–46 yo	No	Overnight fasting + 200 mL of water with the administration; standardized food and beverages were provided: breakfast at 1 h time point, lunch after 5 h time point, and dinner after 8 h time point. No further food allowed except water	Formulation Control	307 mg curcumin 307 mg curcumin	2,274,000 ^c,4^ 49.87 ^c,4^	[66]
Curcuminoids + fenugreek dietary fibers	CurQfen^®^	Randomized double-blind crossover	50 43 males 7 females 24-46 yo	No		Formulation Control	76.7 mg curcumin 76.7 mg curcumin	963 ^c,4^ 38.8 ^c,4^	
Lipid droplet micromicellar technique	Biocurc^®^	Randomized open label parallel	20 males 26.45 ± 5.02 yo	No	10 h overnight fasting + 4 h fasting post administration. Curcumin- and garlic-free meals were provided	Formulation	750 mg curcumin	231.31 ± 24.45 ^a,5^	[82]
Lipid droplet micromicellar technique	Biocurc^®^	Randomized double-blind crossover	12 6 males 6 females 18–45 yo	No	-	Formulation Control	64.6 mg curcumin 323 mg 95% curcumin	351 ^b,6^ 4.16 ^b,6^	[63]
Nano-particle colloidal dispersion	Theracurmin^®^	Randomized	14 8 males 6 females 30–59 yo Japanese	Yes	Administration with 100 mL of water	Formulation Control	30 mg curcumin 30 mg curcumin	113 ± 61 ^c,2^ 4.1 ± 7 ^c,2^	[61]
Nano-particle colloidal dispersion	Theracurmin^®^	Single-dose, double-blind, 4-way crossover	24 13 males 11 females 23–32 yo; Japanese	Yes	Overnight fasting and light lunch after assumption. Administration such as nutritional beverage	Formul. A Formul. B Formul. C Formul. D	30 mg curcumin 30 mg curcumin 40 mg curcumin 30 mg curcumin	121 ± 65.6 ^c,3^ 79.5 ± 31.4 ^c,3^ 40.9 ± 24.9 ^c,3^ 30.1 ± 14.4 ^c,3^	[59]
Natural turmeric matrix obtained by polar-nonpolar sandwich technique	Cureit^®^	Pilot, crossover	12 males (18–45 y)	No	Overnight fasting for at least 8 h	Formulation Control	190 mg curcumin 500 mg curcumin	980 ± 508.104 ^c,7^ 192.8 ± 63.886 ^c,7^	[83]
Natural turmeric matrix obtained by polar-nonpolar sandwich technique	Cureit^®^	Randomized open-label three treatment groups, and parallel comparative design	45 males 18–45 yo	No	10 h overnight fasting, 4 h fasting post administration with 250 mL of water. Lunch, snacks, and dinner at 4, 8, and 12 h from the time of dosing	Formulation Volatile oil formulation Lecithin formulation	180 mg curcumin 351 mg curcumin 80.5 mg curcumin	824.9 ± 466.5 ^c,5^ 117.3 ± 56.8 ^c,5^ 187.3 ± 190.9 ^c,5^	[84]
Lecithin formulation	Meriva^®^	Randomized double-blind crossover	9 8 males 1 female 35 ± 10 yo	Yes	Overnight fasting before administration followed by a standardized breakfast and lunch after 4 h of blood draw (one plain bagel with cream cheese). Subjects were released after the 8 h blood draw, resuming their normal dietary intake but restricting foods with curcumin	Formulation high Formulation low Control	297 mg curcumin 165 mg curcumin 1295 mg curcumin	538.0 ± 130.75 ^e^ 272.6 ± 68.52 ^e^ 122.5 ± 29.35 ^e^	[60]
Solid lipid particle formulation	Longvida^®^	Single-dose double-blind crossover	6 males 18–40 yo Indians	No	10 h fasting prior to administration, 1 h fasting post administration. Administration with 240 mL of water	Formulation Control	130-–195 mg Curcumin >390 mg curcumin	95.26 ± 4.62 ^a,5^ NA	[85]

Control: unformulated curcumin; ND: not detected; AUC: area under the drug concentration–time curve; NA: not applicable; ^a^ mean ± standard error of mean; ^b^ mean; ^c^ mean ± standard deviation; ^d^ no reported standard deviation or error; ^e^ results in mg/mL; ^1^ AUC_0-3_; ^2^ AUC_0-6_; ^3^ AUC_0-8_; ^4^ AUC_0-12_; ^5^ AUC_0-24_; ^6^ AUC_0-48_; ^7^ AUC_0-∞_.

**Table 2:** Clinical studies of curcumin considering various curcumin-based formulations.

Commercial Product	Study Design	Clinical Trial Number	Subjects	Disease	Dose/Intervention	Time	Clinical Trial Results	Refs.
Curcumin C^3^ Complex^®^ + Bioperine^®^	Randomized controlled parallel-group	UMIN Clinical Trials No. UMIN000033774	70 (treatment *n* = 35 46.63 ± 2.2 yo; placebo *n* = 35 47.51 ± 2.4 yo Asians)	NAFLD	500 mg Curcumin C^3^ Complex^®^ + 5 mg Bioperine^®^ after meal	12 weeks	↓ ALT (*p* = 0.035), AST (*p* = 0.042), ALP (*p* = 0.004) in the curcumin + piperine group ↑ ALB and LDH (*p* = 0.001) in the placebo group ↓ cholesterol (*p* < 0.016) and LDL-C (*p* < 0.017) in the curcumin + piperine group ↓ cholesterol (*p* = 0.035) and LDL-C (*p* = 0.000) in the placebo group ↓ NAFLD severity (*p* < 0.001) in the curcumin + piperine group *No adverse effects on hematological parameters*	[73]
Curcumin C^3^ Complex^®^ + Bioperine^®^	Randomized double-blind placebo-controlled	-	49 (18–70 yo Asians)	NAFLD	500 mg Curcumin C^3^ Complex^®^ + 5 mg Bioperine^®^ after breakfast	8 weeks	Weight changes at baseline and after intervention in the curcumin + piperine compared to placebo (*p* = 0.016) ↓ serum concentrations TNFα (*p* = 0.001), EGF (*p* = 0.0001), MCP-1 (*p* = 0.008) in the curcumin + piperine group *NAFLD improvement by curcumin + piperine supplementation*	[74]
BCM-95^®^CG Biocurcumax™	Randomized non-inferiority controlled	Clinical Trials Registry India CTRI/2017/02/007962	144 37 males 107 females; BCM-95^®^CG group *n* = 73 53.1 yo; paracetamol group *n* = 71 50.8 yo)	Knee OA	500 mg BCM-95^®^CG caps twice a day or 650 mg paracetamol tablet thrice a day	6 weeks	↓ TNFα in turmeric compared to paracetamol (*p* = 0.0095) ↓ WOMAC pain score and WOMAC pain and function/stiffness score *All patients with knee OA acheived ≥ 20% score decreases; 18% of patients with knee OA in the turmeric group got ≥ 50% increase in WOMAC pain and function*/*stiffness score; 3% acheived ≥ 70% improvement; no patients in the paracetamol group acheived ≥ 50% improvement (18% vs 0%; p = 0.0002)*	[79]
BCM-95^®^CG Biocurcumax™	Prospective randomized open-label parallel-group	ISRCTN registry ISRCTN 10074826	140 98 males 42 females BCM-95^®^CG + diclofenac group *n* = 71; diclofenac group *n* = 69 38–65 yo	Knee OA	50 mg of diclofenac twice daily or 500mg BCM-95^®^CG + 50 mg diclofenac twice a day Patients were provided with paracetamol 500 mg tab and Ranitidine 150 mg tab as rescue medication	4 weeks	Patients receiving BCM-95^®^CG + diclofenac showed larger improvement in symptoms, pain, and QoL than those treated only with diclofenac (*p* < 0.001) ↓ pain intensity in patients treated with BCM-95^®^CG + diclofenac than those treated with diclofenac alone (*p* < 0.001) Lower number of patients who required H_2_ blockers in the BCM-95^®^CG + diclofenac group compared to the diclofenac group (6% *vs* 28%, respectively; *p* < 0.001) Lower need for rescue medication (*p* < 0.005) in the BCM-95^®^CG + diclofenac group (3%) compared to the diclofenac group (17%) *13% of patients treated by curcuminoid complex + diclofenac and 38% of patients treated with diclofenac showed undesirable effects (p < 0.001)*	[80]
Novasol^®^	Randomized double-blind crossover	Registered at clinicaltrials.gov (NCT01925547)	42 17 males 50 ± 20 yo; 25 females 52 ± 16 yo	Moderate hyperlipidemia - metabolic syndrome risk	80.4 mg of curcumin thrice a day with each principal meal (241.2 mg curcumin/day) or placebo	6 weeks	TC, TAG, HDL-C, LDL-C, and LDL/HDL-C concentrations neither diverged among treatments nor showed a difference over time CRP and IL-6 concentrations were neither influenced by treatment nor time A time effect (*p* < 0.001), but no treatment effect or treatment × time interaction, was detected for fasting glucose concentration Free iron, transferrin, and transferrin saturation were neither modified by treatment nor time; no treatment or time effects were found for ALT and AST activities	[50]
Novasol^®^	Prospective	Registered at clinicaltrials.gov (NCT01712542)	10 4 females 6 males 67 ± 8 yo	GB	1 g of micellar curcumin (57.4 mg curcumin) mixed in 200 mL of pear juice 3 times/day, after meals	4 days	Mean intratumoral concentrations of curcumin, 56 pg/mg tissue (range 9–151) The concentration of curcumin within the tumor correlated with the cumulative dose that patients received (*p* = 0.025). Mean serum concentration of total curcumin was 253 ng/mL (range 129–364)	[88]
CurQfen^®^	Double-blinded randomized placebo-controlled	-	22 males 18–35 yo	Obesity	500 mg CurQfen^®^ or placebo	12 weeks	↓ plasma homocysteine concentrations in the curcumin group compared with the placebo group (*p* = 0.04) ↑ HDL serum concentrations in the curcumin group compared with the placebo group (*p* = 0.04) *No relevant variation was detected in endothelial function between curcumin and control groups*	[91]
CurQfen^®^	Randomized placebo-controlled pilot study	-	22 males 18–35 yo	Obesity	500 mg CurQfen^®^ or placebo	12 weeks	↓ brachial pulse pressure in the curcumin group compared with the placebo group (*p* = 0.040) ↑ anti-inflammatory cytokine IL-10 with curcumin treatment (*p* = 0.071) Different response among the curcumin group detected in cfPWV: responders were subjects with reductions in cfPWV after 12-week curcumin treatment, while the non-responders showed no decreases in cfPWV from baseline rates *The global variation in cfPWV among the curcumin responders shows a larger curcumin-induced destiffening effect for those with increased baseline cfPWV (p = 0.006)*	[92]
Theracurmin^®^	Randomized double-blind placebo-controlled parallel-group	Registered at UMIN UMIN000007361	33 placebo group *n* = 18 14 males 4 females 69 ± 7 yo; treatment group n=15 9 males 6 females 70 ± 6 yo	Impaired glucose tolerance or non-insulin-dependent diabetes mellitus	180 mg of Theracurmin^®^ or placebo	6 months	↑ BS and AT-LDL (*p* = 0.017, *p* = 0.024, respectively) in patients treated by placebo; no significant change in Theracurmin^®^ patients ↓ TG and γ-GTP (*p*= 0.015, *p* = 0.007, respectively) in patients receiving Theracurmin^®^ Time-dependent increase in AT-LDL levels detected in the placebo group (*p* = 0.017) No time-dependent modifications in AT-LDL levels in the Theracurmin^®^ group compared with the placebo group, *Theracurmin^®^ patients showed a higher percentage variation in their BMI. The modification in adiponectin, was negative in placebo patients, while it was positive in Theracurmin^®^ patients*	[95]
Theracurmin^®^	Open-label Prospective	-	45 11 males 34 females 42–85 yo	Knee OA	180 mg of Theracurmin^®^ + combined therapies (NSAIDs, pain relief patches, and hyaluronic acid knee injection treatment) or 180 mg of Theracurmin^®^ (*n* = 13 patients)	6 months	VAS, JKOM, and JOA scores were significantly better after 6-month treatment (*p* < 0.0001, *p* = 0.0003, *p* < 0.0001 respectively) JOA score of 13 Theracurmin^®^ patients considerably improved after the therapy (*p* = 0.0203) *Of the 45 patients, 34 were effective (75.6%) and 11 not effective cases, 8 of which did not feel the efficacy of Theracurmin^®^. The Theracurmin^®^-only group was comprised of 10 effective cases (76.9%) and 3 not-effective cases, 2 of which did not feel the efficacy of Theracurmin^®^*	[96]
Cureit^®^	Randomized, placebo-controlled, double-blind	Registered with Clinical Trials Registry India (CTRI/2018/05/014174)	30 12 males 18 females 36 ± 11 yo	DOMS and related muscle damage	500 mg of Cureit^®^ or placebo	12 weeks	↓ VAS score in subjects treated with Cureit^®^ (*p* < 0.0001) Substantial changes (*p* < 0.001) in the pain score of Cureit^®^ and placebo groups	[100]
Meriva^®^	Observational study	-	124 61 males 63 females 56.4 ± 5.2 yo *n* = 63 Meriva^®^ + glucosamine *n* = 61 chondroitin sulphate + glucosamine	Knee OA	1 tablet/day containing 500 mg Meriva^®^ + 500 mg Regenasure^®^ (vegetarian glucosamine HCl) or 2 capsules/day, containing 400 mg chondroitin sulphate and 415 mg glucosamine HCl	4 months	↑ Karnofsky Index and WOMAC scores in the Meriva^®^ + glucosamine group compared to the chondroitin + glucosamine group ↑ of walking distance on the treadmill in the Meriva^®^ + glucosamine group compared with the baseline and chondroitin + glucosamine group already at 1 month; this benefit was continued until the end of the investigation ↓ need for associated drugs and medical care in both groups: the usage of Meriva^®^ + glucosamine was related to a diminished need for medicines and medical care compared to chondroitin + glucosamine	[102]
Meriva^®^	Pilot study	-	50 males *n* = 25 common analgesic drugs group 18–40 yo *n* = 25 Meriva^®^ group 17–52 yo	osteo-muscular pain	1 tablet of Algocur^®^ every 12 h (each tablet contains 1 g of Meriva^®^) or conventional analgesic drugs	10 days	↓ perceived pain was scored by VAS compared to baseline in both treated groups, starting from the third day of therapy Improvement of impaired physical function assessed in subjects treated with the Meriva^®^-based formulation and in subjects cured with common analgesic drugs, compared to the condition at baseline Adherence to therapy was higher in the Meriva^®^ group (24.96%) compared to the conventional analgesic drug group (15.6%) (*p* = 0.005) *Tolerability scored as outstanding by 24 (96%) subjects treated by the Meriva^®^-based formulation and by 14 (56%) subjects treated with standard therapy (p =0.002)*	[103]
Meriva^®^	Pilot study	-	12 eyes from 11 patients 7 males 4 females	Chronic diabetic macular edema	500 mg of Meriva^®^ twice a day	3 months	BCVA improved after 3 months of treatment 0% of eyes exhibited a decrease in visual acuity, 17% revealed stabilization, and 83% demonstrated improvement (*p* = 0.0072) 92% of eyes revealed a decrease in macular edema, 8% displayed stabilization, and 0% demonstrated a clinically meaningful increase Mean macular thickness decreased from baseline after 3-month treatment (*p* = 0.0090) *58% of eyes showed an improvement for near best-corrected visual acuity and 42% showed stability for near best-adjusted visual acuity*	[104]
Meriva^®^	Prospective single arm single center phase II trial	-	44 29 males 15 females 42–87 yo	Advanced or metastatic pancreatic cancer	2000 mg/day of Meriva^®^ + GEM was infused on days 1, 8, and 15, within a 28-day treatment cycle	42 months	Limited response in 27.3% of patients, stable disorder in 34.1%, progression in 17 patients (38.6%) ↑ basal levels of IL-6 (*p* = 0.03), sCD40L (*p* = 0.05), and CRP (*p* = 0.03) in patients not responding to therapy In responsive patients, no relevant changes in biomarkers were detected among baseline levels and those receiving 1 or 3 cycles of chemotherapy *A total number of 35 patients (79.5%) achieved the baseline QoL questionnaire and 30 (68.2%) filled it in for at least three cycles*	[105]

ALB: albumin; ALP: alkaline phosphatase; ALT: alanine aminotransferase; AST: aspartate aminotransferase; AT-LDL: a1-antitrypsin-LDL; BCVA: best-corrected visual acuity; BMI: body mass index; BS: blood sugar; cfPWV: carotid-femoral pulse wave velocity; CRP: C-reactive protein; DOMS: delayed onset muscle soreness; EGF: epithelial growth factor; GB: glioblastoma; HDL-C: high-density lipoprotein cholesterol; IL-6: interleukin-6; JKOM: Japanese Knee Osteoarthritis Measure; JOA: Japanese Orthopedic Association; Knee OA: knee osteoarthritis; LDH: lactate dehydrogenase; LDL-C: low-density lipoprotein cholesterol; NAFLD: nonalcoholic fatty liver disease; NSAIDs: non-steroidal anti-inflammatory drugs; QoL: quality of life; sCD40L: soluble CD40 ligand; TAG: triacylglycerols; TC: total cholesterol; TG: triglycerides; TNFα: tumor necrosis factor alpha; VAS: visual analog scale; WOMAC: Western Ontario and McMaster Universities’ Arthritis Index; γ-GTP: γ-glutamyl transpeptidase; GEM: gemcitabine.

**Table 3:** A detailed step-by-step description of the DbD approach with an overview of curcumin-fortified beverages fabrication.

Stage Process	Property	Requirements	Curcumin-Fortified Functional Beverage
***Stage 1*** Active agent definition	Molecular and physico-chemical features Solubility features Stability features	→ *Chemical formula and structure, molar mass, molecular dimensions, density, refractive index, melting and boiling points, partitioning, diffusion coefficient, molar volume, surface tension, solubility, pK_a_ values, hydrogen bond acceptors*/*donors* → *Molecular weight, number of hydrogen bond donors*/*acceptors, oil-water partition coefficient, ionic strength, pH, matrix composition, temperature* → *pH, temperature, oxygen levels, pro-oxidant levels, light exposure*	▪ Polyphenol, molecular formula: C_21_H_20_O_6_ ▪ Low-molecular-weight substance (368.380 Da) ▪ At room temperature, it exits as a yellow-orange crystallized powder (melting point 180 °C, boiling point 521.3 °C). Density 1.3 g/cm, refractive index 1.643 ▪ Water solubility is highly pH-dependent (24 mg/L at pH < 8, while it becomes hydrophilic at pH > 11) and its surface and interfacial tension (54.3 dyne/cm) ▪ Oil solubility tends to increase as the molecular weight of the carrier oil decreases—olive oil (1.18 mg/mL), corn oil (3 mg/g), tributyrin (29.8 mg/g), medium-chain triglycerides (7.9 mg/g), sunflower oil (1.08 mg/mL) ▪ The partition coefficient oil/water is expressed as LogP value (4.12), influenced by pH. pH values could influence electrical charge thanks to the presence of ionizable functional groups (pKa values of the three readily dissociable hydrogen atoms: 8.1, 9.5, and 10.1) ▪ 6 O_2_ atoms act as hydrogen bond acceptors, while 2 H_2_ atoms can donate hydrogen bonds ▪ Highly stable at acidic condition (half-life around 175 days at pH 5.97) but the degradation rate increases at the increase in pH values ▪ Photochemical degradation occurs more frequently under visible light than UV light ▪ Thermal degradation occurs at high temperature ▪ Dissolution in oils prevents degradation compared to dissolution in water
***Stage 2*** End-product definition	Compositional analysi Environmental stress analysis Physical state and rheological properties Optical properties	→ *pH, ionic strength, polymer type, transition metals, surface-active substances* → *Temperatures, oxygen levels, light conditions* → *Solid, semi-solid, liquid, viscosity, elastic modules, breaking stress* → *Transparency, color*	▪ The product pH should be 6.3-–6.8 with total carbohydrates ≤10%, including dietary fibers ≥0.25%, total fat ≤5%, protein ≥3%, minerals (ionic strength of 90–110 mM) ▪ Curcumin content should be at least 150 mg/100 g of product and the quantity reaching the blood should exceed 50 ng/mL ▪ It would be desirable to use plant-based ingredients to meet the favor of health-conscious consumers ▪ The product should be physically stable and avoid phase separation or increase in mean particle size as well as chemically stable: curcumin content should not decrease by >15% and color should not reduce during manufacturing, storage, and consumption (by >5% at 20 °C or by >10% at 30 °C for 6 months, in light exposure) ▪ The product should stay stable after heating (72 °C for 15 s) and at temperatures 0–45 °C for 12 months of storage, and after exposure to high-shear mixing conditions (500 rev/min for 5 min) ▪ Ideally, the product should be like the “golden milk”, a creamy, cloudy, and yellow beverage (viscosity of 2–4 mPa·s) with an earthy spicy flavor profile
***Stage 3*** Delivery system specification	Physical form, rheological properties Optical properties Stability characteristics Functional attributes	→ *Solid, semi-solid, liquid, viscosity, elastic modules, breaking stress* → *Clear, turbid, or opaque* → *pH values, temperatures, ionic strengths, light exposure, oxygen levels, ingredients* →* Loading, active agent protection, retention, and release profile, dispersibility*	▪ Fluid or powdered delivery systems would readily disperse into an aqueous solution at room temperature (pH 6.5) ▪ The delivery system should match the appearance of the end product that is a creamy yellow aspect ▪ Colloidal particles should remain stable to aggregation, growth (increase >20% of particle diameter), dissolution, or separation during storage ▪ Physical and chemical stability should be maintained after thermal processing at 75 °C for 15 s, when stored at temperatures of 0–45 °C (12 months), or at pH values ranging from 6.0 to 7.0 for 12 months (30 °C) ▪ Curcumin not degraded (*<*20%) during production, storage, and utilization; concentration of active curcumin should be >1000 mg when stored for 12 months (30 °C, pH 6.5) ▪ The delivery system should prevent curcumin degradation during passage through certain parts of the GI tract, while allowing release in others
***Stage 4*** Particle specification & delivery system selection	Particle characteristics Loading characteristics Retention and release characteristics Environmental responsiveness Delivery system selection	→*Particles, composition, concentration, morphology, dimensions, physico-chemical properties, charge interactions* → *Loading capacity, encapsulation efficiency* → *Prolonged, burst, or triggered release* → *Ease of manufacture, cost, legal status, and consumer acceptability (plant-based*/ *vegetarian, animal-based, Kosher, or synthetic ingredients)* → *Micelles, liposomes, emulsions, microemulsion, nanoemulsion, solid lipid nanoparticles, or polymer particles*	▪ To obtain a creamy beverage stable to gravitational separation, colloidal particles should be relatively small (*d* < 300 nm) and/or have great density, have a smooth mouth feel, and the diameter of the colloidal particles should be smaller than 50 μm ▪ Since curcumin is a hydrophobic molecule, it would be advantageous to use colloidal particles with a hydrophobic interior; moreover, curcumin has higher solubility in medium-chain triglycerides (e.g., coconut oil) and improved bioaccessibility in MCT-nanoemulsions ▪ The delivery system should be designed to selectively break down in the small intestine and release free fatty acids for increasing the curcumin solubilization capability within the intestinal liquids ▪ The ideal delivery system should be the simplest and cheapest (lower than 2 cents per unit) to manufacture; ingredients should preferably be plant-based or derived from natural food-grade components ▪ Among the variety of the delivery systems available, oil-in-water emulsions and nanoemulsions are the most appropriate delivery system for curcumin, thanks to their ease of fabrication with plant-based emulsifiers and lipids, their optically opaque appearance, and their low viscosity. They have great stability and a superior bioavailability
***Stage 5*** Process specification	Ingredient quality: Critical ingredient attributes Manufacturing steps: Critical processing attributes	→ *Composition, quality; ingredient properties*/*colloidal delivery system performance interaction* → *Resources, equipment, and facilities available; cost, simplicity, reliability, scale up; dissolution times, temperatures, mixing conditions, appropriate particle size distribution.*	▪ According to the equipment, resources, and available facilities, different approaches to produce colloidal particles required for the end-product are available ▪ To prepare nanoemulsions, it is possible to dissolve curcumin in the oil phase and then homogenize with an aqueous protein mixture, employing a high-pressure homogenizer or microfluidizer. Experiments should be performed for optimizing the system composition and homogenization conditions (minimum mixing time and mixing rate, optimum solubilization temperature, protein concentration, and operating pressure/number of passes through the homogenizer for obtaining nanoemulsions with the desirable droplet size) ▪ Otherwise, nanoemulsions could be produced thanks to the pH-driven method, which consists of loading curcumin into a preexisting nanoemulsion or milk-like product starting from an alkaline curcumin solution mixed with an acidic colloidal dispersion at room temperature
***Stage 6*** Performance testing	Analytical tools Testing protocols	→ *Static and dynamic light scattering techniques, microelectrophoresis, optical, confocal fluorescence, electron microscopy, colorimetry, spectrophotometry, chromatography, mass spectrometry, sensory tests* → *In-product tests, accelerated screening tests, environmental stress tests*	▪ The particle size distribution of the nanoemulsions and their electrical characteristics (*ζ* potential) could be measured by dynamic light scattering and particle electrophoresis, respectively ▪ Environmental stress could be affected by exposing it to ionic strength, different pH and temperature, and mechanical conditions ▪ Measuring the optical properties (colorimeter), rheology (rheometer), and phase separation (visual observation) of the functional beverage allows researchers to monitor the impact of the delivery system on the appearance, texture, and stability of the end-product
***Stage 7*** System optimization	Monitoring and tabulating the characteristics of the delivery system and end-product throughout its life cycle so as to optimize their properties and make appropriate adjustments where possible, if needed		

## Abbreviations

3D	Three dimensional
γ-GTP	γ-glutamyl transpeptidase
Akt	Protein kinase B
ALB	Albumin
ALP	Alkaline phosphatase
ALT	Alanine aminotransferase
AST	Aspartate aminotransferase
AT-LDL	A1-antitrypsin-LDL
AUC	Area under the curve
Bax	Bcl-2-like protein 4
BCS	Biopharmaceutics classification system
BCVA	Best-corrected visual acuity
BMI	Body mass index
BS	Blood sugar
CAu-PLGA NPs	Gold-loaded PLGA nanoparticles
CCDC	Cambridge Crystallographic Data Centre
cfPWV	Carotid-femoral pulse wave velocity
CK	Creatine kinase
CMC	Critical micellar concentration
CNS	central nervous system
CRP	C-reactive protein
CYP3A4	Cytochrome P450 3A4
DbD	Delivery by Design
DOMS	Delayed onset muscle soreness
EGF	Epithelial growth factor
FAE	Follicle-associated epithelium
FDA	US Food and Drug Administration
GALT	Gut-associated lymphoid tissue
GB	Glioblastoma
GEM	Gemcitabine
GI	Gastrointestinal
GRAS	Generally regarded as safe
HDL	High-density lipoprotein
HDL-C	High-density lipoprotein cholesterol
IκBα	nuclear factor of kappa light polypeptide gene enhancer in B-cells inhibitor α
IL-1β	Interleukin-1β
IL-6	Interleukin-6
i.p.	Intraperitoneal
JKOM	Japanese knee osteoarthritis measure
JOA	Japanese orthopedic association
LDH	Lactate dehydrogenase
LDL	Low-density lipoprotein
LSCS-CUR	Lauroyl sulphated chitosan–curcumin
M cells	Microfold cells
MCT	Medium-chain triglyceride
MNPs	Magnetic nanoparticles
mPa·s	Millipascal second
NAFLD	Nonalcoholic fatty liver disease
NF-kB	Nuclear factor kappa-light-chain-enhancer of activated B cells
Nrf2	Nuclear factor erythroid 2–related factor 2
NSAIDs	Non-steroidal anti-inflammatory drugs
OCT	Optical coherence tomography
PARP	Poly (ADP-ribose) polymerase
PEG	Polyethylene glycol
PEG 400	Polyethylene glycol 400
PEO	Polyethylene oxides
PNS	Polar–nonpolar sandwich
PPO	Polypropylene oxide
PPs	Peryer’s patches
PLGA	Poly-(lactic-co-glycolic acid)
PVP	Polyvinyl pyrrolinone
QoL	Quality of life
OA	Osteoarthritis
ROS	Reactive Oxygen Species
sCD40L	Soluble CD40 ligand
SMEDDSs	Self-microemulsifying drug delivery systems
SLCPs	Solid lipid curcumin particles
SLNPs	Solid lipid nanoparticles
SLPs	Solid lipid particles
TAG	Triacylglycerols
TC	Total cholesterol
Tf	Transferrin
TG	Triglycerides
TNFα	Tumor necrosis factor α
TNFβ	Tumor necrosis factor β
UDP-glucuronosyltransferase	Uridine 5′-diphospho-glucuronosyltransferase
UMIN Clinical Trials	University hospital medical information network Clinical Trials
UV light	Ultraviolet light
VAS	Visual analog scale
WOMAC	Western Ontario and McMaster Universities’ arthritis index
